# Does epigenetic polymorphism contribute to phenotypic variances in *Jatropha curcas *L.?

**DOI:** 10.1186/1471-2229-10-259

**Published:** 2010-11-23

**Authors:** Chengxin Yi, Shilu Zhang, Xiaokun Liu, Ha TN Bui, Yan Hong

**Affiliations:** 1 JOil (S) Pte Ltd, 1 Research Link, Singapore 117604; 2 Temasek Life Sciences Laboratory, 1 Research Link, National University of Singapore 117604

## Abstract

**Background:**

There is a growing interest in *Jatropha curcas *L. (jatropha) as a biodiesel feedstock plant. Variations in its morphology and seed productivity have been well documented. However, there is the lack of systematic comparative evaluation of distinct collections under same climate and agronomic practices. With the several reports on low genetic diversity in jatropha collections, there is uncertainty on genetic contribution to jatropha morphology.

**Result:**

In this study, five populations of jatropha plants collected from China (CN), Indonesia (MD), Suriname (SU), Tanzania (AF) and India (TN) were planted in one farm under the same agronomic practices. Their agronomic traits (branching pattern, height, diameter of canopy, time to first flowering, dormancy, accumulated seed yield and oil content) were observed and tracked for two years. Significant variations were found for all the agronomic traits studied. Genetic diversity and epigenetic diversity were evaluated using florescence Amplified Fragment Length Polymorphism (fAFLP) and methylation sensitive florescence AFLP (MfAFLP) methods. Very low level of genetic diversity was detected (polymorphic band <0.1%) within and among populations. In contrast, intermediate but significant epigenetic diversity was detected (25.3% of bands were polymorphic) within and among populations. More than half of CCGG sites surveyed by MfAFLP were methylated with significant difference in inner cytosine and double cytosine methylation among populations. Principal coordinates analysis (PCoA) based on Nei's epigenetic distance showed Tanzania/India group distinct from China/Indonesia/Suriname group. Inheritance of epigenetic markers was assessed in one F1 hybrid population between two morphologically distinct parent plants and one selfed population. 30 out of 39 polymorphic markers (77%) were found heritable and followed Mendelian segregation. One epiallele was further confirmed by bisulphite sequencing of its corresponding genomic region.

**Conclusion:**

Our study confirmed climate and practice independent differences in agronomic performance among jatropha collections. Such agronomic trait variations, however, were matched by very low genetic diversity and medium level but significant epigenetic diversity. Significant difference in inner cytosine and double cytosine methylation at CCGG sites was also found among populations. Most epigenetic differential markers can be inherited as epialleles following Mendelian segregation. These results suggest possible involvement of epigenetics in jatropha development.

## Background

Since the petroleum crisis in 1970 s and the recognition of limited world fossil energy resources, plant oils which can replace fossil oil have been given more attention [1]. Special interest has been shown in the cultivation of *Jatropha curcas *(jatropha, a member of Euphorbiaceae family) for its drought and poor soil tolerance. It can be cultivated on marginal land and does not compete with food production crops. Jatropha, also known as physic nut, is a small tree or large shrub which can reach a height of 5 m [2]. The plant is monoecious and the flowers are unisexual, occasionally hermaphrodite flowers occur. Pollination of the jatropha is by insects because its sweet, heavy perfume, greenish white flowers, versatile anthers and protruding sexual organs, copious nectar, and absence of visible nectar guides [3]. During field trials, it was observed that a number of different insects visited the flowers and helped in pollination. When insects are excluded from the greenhouse, seed set does not occur without hand-helping pollination. It is speculated that the center of origin of jatropha is Central America. From the Caribbean, this species was probably distributed by Portuguese seafarers via the Cape Verde Islands and former Portuguese Guinea (now Guinea Bissau) to other countries in Africa and Asia [2].

The understanding of biodiversity (both phenotypic and genetic diversity) of jatropha is essential to developing strategies for collection, conservation and new variety development. Before 1996, few systematic provenance trials had been conducted to exam morphological characteristic differences among various jatropha collections. All except one found out clear phenotypic differences among collections (summarized by Heller 1996) [2]. For example, a trial found out a good degree of variation in plant height, branches per plant, branched habit, leaves and seeds size and seed yields between India and Nicaragua collections [2]. Recently, Kaushik [4] assessed the variability in seed traits and oil content of 24 accessions of jatropha collected from different agroclimatic zones of Haryana state, India and revealed that there were significant differences in seed size, 100-seed weight and oil content among accessions. There have been many efforts to access the extent of genetic diversity in jatropha by using various molecular markers. Basha et al reported low degree of variation among the accessions collected from different geographical regions of India [5]. In their further research [6], genetic background of 72 jatropha accessions representing 13 countries were elucidated using molecular analysis and biochemical analysis. The biochemical composition analysis of seeds showed wide variation in crude protein, oil and ash content and phorbol ester in seed. However, simple sequence repeat (SSR) analysis found out that accessions from many countries failed to be distinguished with the exception of accessions from Mexico and El Salvador. A recent study [7] used RAPD, AFLP and cTBP to assess genetic polymorphism in *J. curcas *accessions from 13 countries on 3 continents. High degree of monomorphism in *J. curcas *accessions was found among the collections, only the accessions from Mexico and Costa Rica exhibited polymorphism. Overall, clear phenotypic variation among jatropha collections is well established. However, high phenotypic variation is matched by low level of genetic diversity as revealed by molecular markers.

Methylation of cytosine is an important epigenetic modification of the nuclear DNA of many eukaryotic organisms. Although these changes do not alter the primary DNA sequence, they are frequently heritable through cell division, sometimes for multiple generations and can thus often be classified as epigenetic marks [8]. These conserved epigenetic marks have been found to influence many aspects of gene expression and chromosome biology, and they have characteristic genomic distribution [9]. Epigenetic changes can occur at a high frequency in crop plants and might generate phenotypic variation that is not correlated with genetic variation [10]. In this study, collections from 5 different countries on three continents have been evaluated for phenotypic variation under the same agronomic climate and practices. The important agronomic traits included in our study are: first year yield per plant (1yY), second year yield (2yY), number of primary branch (NPB), number of fruiting branch (NFB), diameter of canopy (DiC) after two years, plant height (H) after two years, oil content in seed (Oil %) after one year, days from sowing to flowering (DtF) and dormancy of the branch shoot. Many of the traits have not been monitored by any previous work. On the other hand, genetic diversity was evaluated by fAFLP, epigenetic diversity was evaluated by methylation sensitive fAFLP (MfAFLP). An intra-species hybrid F1 population and one selfed population were used to evaluate inheritance of epigenetic markers and their pattern of inheritance. One epigenetic allele was further confirmed by bisulphite sequencing.

## Results

### Phenotypic variances between different populations

Randomly selected continuous 10 individuals from each population were tracked for the following agronomic traits: first year yield per plant (1yY), second year yield (2yY), number of primary branch (NPB), number of fruiting branch (NFB), diameter of canopy (DiC) after two years, plant height (H) after two years, oil content in seed (Oil %) after one year, days from sowing to flowering (DtF) and dormancy of the branch shoot at drough periods. Significant variations of agronomic traits were observed in the five populations studied (Table 1). First flowering time ranged from 105 days for MD and 175 days for TN, a difference of around 70 days. Accumulated yield for the first year (1yY) varied greatly among population, with an average of 370 grams per tree for MD and as little as 25 grams per tree for CN, a difference of more than 10 times. Such yield difference continued but narrowed down in the second year, with 708 grams per tree for MD and 217 grams per tree for CN. There were also significant differences in plant height and tree canopy size: with AF being the tallest population and MD being the population with largest canopy. Primary branches as well as fruiting branches were most numerous in SU population. In the very hot and dry months, apical bud dormancy was observed but with different severity: most serous in TN and CN populations but much less in MD population. Seed oil content was also variable among populations with AF population had the highest oil content and CN had the lowest oil content. Overall, the population from Indonesia was the earliest to flower and the most productive for both the first and the second year.

**Table 1:** Agronomic trait variations among populations (average ± SE)

Pops	1yY (g)	2yY (g)	H (cm)	DiC (cm)	NFB	NPB	Dormancy	Oil (%)	DtF (d)
MD	370.7 ± 43.2 A	708.0 ± 59.2 A	193.0 ± 3.4 B	217.0 ± 3.9 A	30.1 ± 3.0 A	6.9 ± 0.7 AB	< 5%	28.0 ± 0.9 AB	105.4 ± 5.2 C
AF	213.9 ± 33.6 B	364.3 ± 36.9 B	219.0 ± 3.3 A	192.3 ± 3.4 BC	21.0 ± 0.8 AB	7.8 ± 0.8 AB	20-30%	31.5 ± 1.2 A	144.3 ± 2.9 B
SU	66.2 ± 10.5 C	330.6 ± 41.3 B	182.9 ± 4.7 BC	172.3 ± 6.0 CD	22.8 ± 2.1 AB	9.4 ± 0.6 A	20-30%	28.1 ± 1.0 AB	139.6 ± 3.7 B
TN	29.8 ± 5.2 C	301.6 ± 45.6 B	180.3 ± 3.1 BC	200.5 ± 5.2 AB	22.6 ± 2.1 AB	8.8 ± 0.4 AB	40-50%	26.0 ± 0.6 B	175.3 ± 7.3 A
CN	24.9 ± 4.5 C	217.4 ± 45.3 B	173.0 ± 2.8 C	162.5 ± 4.5 D	17.7 ± 1.2 B	6.4 ± 0.5 B	40-50%	25.9 ± 0.9 B	150.7 ± 1.3 B

Note: Seed derived jatropha populations: Tanzania, Africa (AF), Indonesia (MD), Yunnan Province, China (CN), Tamil Nadu, India (TN), Suriname, South America (SU); agronomic traits: 1yY, one year seed yield; 2yY: two year seed yield; H: plant height after two years; DiC, diameter of canopy after two years; NFB: number of flowering branches; NPB: number of primary branches; Dormancy: percentage of plants going into dormancy during drought period; Oil: seed oil content: DtF:days from sowing to first flowering; A, B, C: ranking by Tukey analysis with significance of 0.01.

Besides the differences among populations, there were also the differences within populations. The coefficient variation (CV%, calculating SE as a percentage of the average) for traits 1yY, 2yY, NFB and NPB were larger than 15% within population, suggesting big variation of agronomic traits even within the same population.

### Very low level of genetic diversity between and within different populations

Initially 64 primer combinations of E*coR *I/M*se *I with three nucleotides selective primers were screened for fAFLP DNA genotyping. A total of 23 primer combinations with each giving about 25 distinct bands (see Additional file 1 for details of primers), were selected for further analysis. A total number of 575 bands were identified by fAFLP analysis of all 162 individuals, out of which only 3 polymorphic bands were detected in 5 plants from China and India (one representative fAFLP result is given in Additional file 2). This represents that only 0.52% of bands were polymorphic. The experiment was repeated twice with the same result. To further investigate the genetic diversity of jatropha, RAPD, DAMD and SSR primers which other researchers used in their studies were also used to analyze our populations. However, the 22 RAPD primers and 2 SCAR primer pairs used by Basha et al [5] and the 7 RAPD primers and 4 DAMD primers from Shirish et al (2008) [11] and 12 SSR primer pairs from Sudheer et al (2009)[12] all failed to detect a single polymorphism in our samples. AFLP, RAPD and DAMD markers are generally believed to distribute randomly throughout genome, SCAR and SSR have specific distribution patterns. Together, these results suggest little genetic diversity among and within the populations we studied.

### Moderate but significant epigenetic diversity revealed by MfAFLP genotyping

For MfAFLP analysis, 54 primer combinations for E*coR *I/M*sp *I (EM) and E*coR *I/H*pa *II (EH) with three nucleotide selective primers were firstly screened on 5 random selected samples with one from each population. 14 primer combinations (Additional file 1) with the most number of amplified bands were used for further analysis of the same 162 individuals by the genetic diversity analysis. A total of 562 distinct bands differentiating between EM and EH in at least one sample were identified (Representative MfAFLP results are given in Additional file 3 and file 4). The percentage of polymorphic epigenetic band varied from 22.42% in AF population and 27.58% in CN population with the mean percentage of polymorphic bands being 25.30% and mean heterozygosity being 0.062 (Table 2). Only MD population had one private band (Table 2). The AMOVA analysis showed that 28% of the epigenetic variance was assigned to variance among populations and 72% to variance within populations and the differences among and within populations were significant.

**Table 2:** Total epigenetic diversity by jatropha populations

Population	AF	CN	MD	SU	TN	Total
No. of plants	48	30	31	30	23	162
No. Bands	324	347	341	341	340	562
No. Private Bands	0	0	1	0	0	
Mean	0.048	0.074	0.072	0.074	0.043	0.062
Heterozygosity (SE)	(0.005)	(0.006)	(0.006)	(0.006)	(0.005)	
Percentage of polymorphic bands	22.42%	27.58%	27.05%	26.87%	22.90%	25.30%

**Figure 1:**
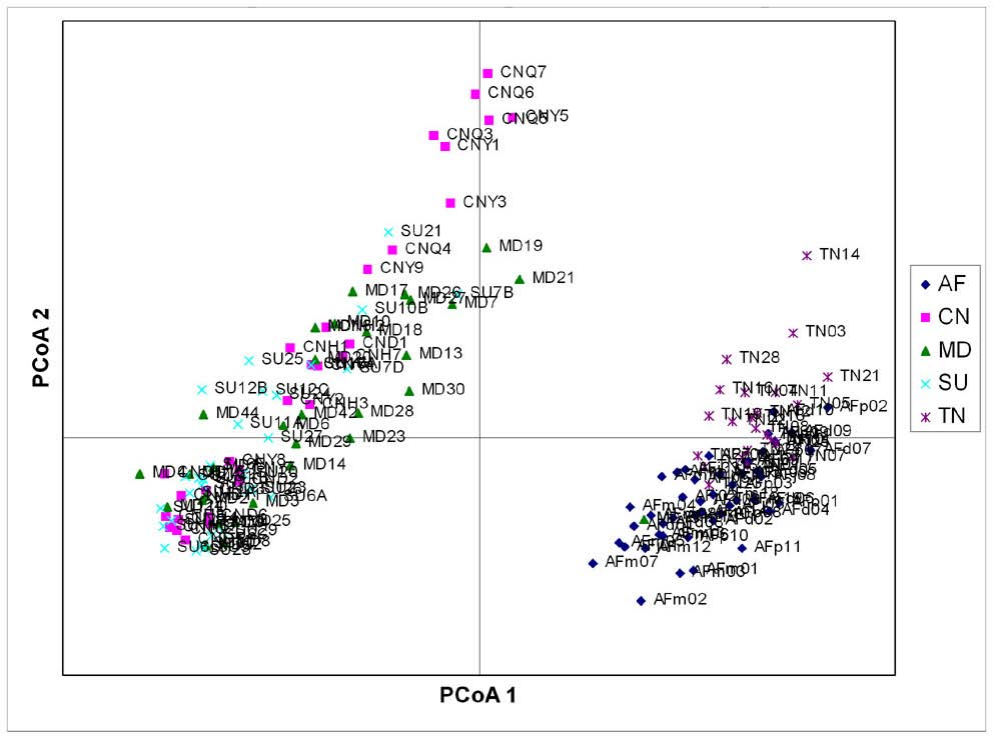
**Principal Coordinates Analysis (PCoA) based on Nei's epigenetic distance**.

Relativeness among the 5 populations was examined by Nei's epigenetic distances analysis and showed in Table 3. Principal coordinates analysis (PCoA) was performed based on the matrix of Nei's epigenetic distances among samples and showed in Figure 1. PCoA analysis revealed two jatropha clusters, one containing AF and TN populations, and the other containing CN, MD and SU populations.

**Table 3:** Nei's epigenetic distance between jatropha populations

AF	CN	MD	SU	TN	
0.000					AF
0.031	0.000				CN
0.029	0.011	0.000			MD
0.035	0.011	0.013	0.000		SU
0.009	0.036	0.035	0.042	0.000	TN

### Variation of CCGG methylation among populations

MfAFLP is a modified version of standard AFLP [13] using a pair of methylation-sensitive restriction enzymes, H*pa *II and M*sp *I, which are a pair of isoschizomers, recognize the same restriction site CCGG but possess differential methylation sensitivity at the inner or outer cytosine [14]. In the absence of cytosine methylation, both enzymes can cut a CCGG site. H*pa *II can't cut when the inner cytosine is methylated. M*sp *I can't cut when the outer cytosine is methylated. Any difference in AFLP banding pattern between E*coR *I + H*pa *II (EH) and E*co*R I + M*sp *I (EM) digested DNAs should reflect different states of cytosine methylation at CCGG sites. We found out that all bands were differentiating EH and EM in at least one sample, indicating absence of genetic variation at all CCGG sites surveyed, hence the MfAFLP result exclusively reflects epigenetic diversity at the CCGG sites.

The ANOVA analysis for the variation of CCGG methylation pattern among 5 populations was conducted based on frequencies of absence of cytosine methylation (non-C^m^), methylation at both inner and outer cytosines (both-C^m^), methylation of inner cytosine only (only-inner-C^m^) and methylation of outer cytosine only (only-outer-C^m^) in each population (Table 4). These multiple comparison tests were based on Tukey (HSD) analysis of the differences between populations with a confidence level of 99%. Nearly half of CCGG sequences were not methylated (non-C^m^), with no significant difference among populations. For the three types of methylation, there were significant differences among populations in inner cytosine and double cytosine methylation. MD, CN and SU are higher for inner cytosine methylation but lower for double cytosine methylation. TN and AF have the exact opposite situations. Inner cytosine methylation and double cytosine methylation together happen in nearly half of CCGG sequences. The single outer cytosine methylation happens in less than 5% of CCGG sequences. Consistent with the PCoA analysis, AF and TN populations form a separate group from the group of MD, SU and CN populations. The high and varied level of CCGG methylation suggests possible role of methylation in gene regulation.

**Table 4:** CCGG methylation in the jatropha populations (average ± SE)

Pops	No. of samples	CC^m^GG	C^m^C^m^GG	C^m^CGG	CCGG
		
		Only-inner-C^m ^(%)	Both-C^m ^(%)	Only-outer-C^m ^(%)	Non-C^m ^(%)
MD	32	33.05 ± 0.54 A	18.04 ± 0.33 B	3.53 ± 0.26 B	45.39 ± 0.60
CN	30	32.26 ± 0.32 AB	17.35 ± 0.61 B	3.48 ± 0.19 B	46.91 ± 0.67
SU	30	31.20 ± 0.41 B	16.89 ± 0.34 B	4.51 ± 0.22 A	47.41 ± 0.52
AF	48	27.28 ± 0.20 C	22.42 ± 0.18 A	4.04 ± 0.11 AB	46.26 ± 0.25
TN	22	27.11 ± 0.34 C	22.46 ± 0.32 A	4.35 ± 0.24 AB	46.09 ± 0.34

Note: A, B, C: ranking by Tukey analysis at significance of 0.01.

### Heritability of Epialleles

To reveal the heritability of epigenetic bands of jatropha, we constructed a crossed pollinated population by controlled pollination in an insect free green house between MD24 and TN02 (MT2402 F1Pop), which were from Indonesia and India, respectively. They were different in many agronomic traits including plant height, branching pattern and total number of flowers in florescence. A self-pollinated population of MD24 (MD24ScPop) was also obtained in the similar way. The 17 individuals of MT2402 F1Pop and 25 individuals of MD24ScPop together with their parents were analyzed with MfAFLP. 37 primer combinations were screened and 19 primer combinations were used in final analysis. 39 polymorphic bands from a total of 555 amplified bands were identified. Among the 39 polymorphic bands, 30 bands segregated in both populations in accordance with Mendelian segregation pattern. Table 5 shows the 30 heritable epigenetic bands, their segregation pattern in the 2 populations, corresponding **χ^2 ^**values, and the deduced epiallele's patterns (Ho for homozygous, or He for heterozygous) in MD24 and TN02. The other 9 polymorphic bands segregated but did not follow Mendelian segregation in one or both populations. For example, bands E1H10-076, E4H9-071 and E4H15-550 had significantly biased segregation in MD24ScPop population. Two bands E1H14-228 and E4H1-400 were not present in parents but appeared in some progenies.

**Table 5:** The heritability of epigenetic bands

Markers	MD24	No. of plants in MD24ScPop		χ^2^(3:1)	Probability	MD24 Ho/He	MD24	TN02	No. of plants in MT2402F1Pop		χ^2^(3:1)	χ^2^(1:1)	Probability	TN02 Ho/He
												
		Band	No-band						Band	No-band				
E1H01-085	1	16	9	1.08	0.5-0.25	He	1	1	17	0				Ho
E1H01-142	1	19	6	0.013	0.95-0.9	He	1	1	11	6	0.491		0.5-0.25	He
E1H01-444	1	20	5	0.12	0.75-0.5	He	1	1	11	6	0.491		0.5-0.25	He
E1H02-376	1	18	7	0.013	0.95-0.9	He	1	0	7	10		0.235	0.75-0.5	Ho
E1H05-486	0	0	25			Ho	0	1	9	8		0	> 0.995	He
E1H09-590	1	25	0			Ho	1	0	17	0				Ho
E1H10-076	1	10	15	14.52	< 0.005	?	1	1	17	0				Ho
E1H10-501	1	16	9	1.08	0.5-0.25	He	1	1	17	0				Ho
E1H15-130	1	25	0			Ho	1	0	17	0				Ho
E1H16-294	0	0	25			Ho	0	1	9	8		0	> 0.995	He
E1H16-331	0	0	25			Ho	0	1	5	12		2.118	0.25-0.1	He
E2H08-270	1	19	6	0.013	0.95-0.9	He	1	0	12	5		2.118	0.25-0.1	He
E2H08-450	1	19	6	0.013	0.95-0.9	He	1	1	11	6	0.49		0.5-0.25	He
E2H09-189	1	17	8	0.333	0.75-0.5	He	1	1	13	4	0.02		0.9	He
E2H11-324	1	19	6	0.013	0.95-0.9	He	1	1	17	0				Ho
E2H14-216	1	22	3	1.613	0.25-0.1	He	1	0	11	6		0.941	0.5-0.25	Ho
E4H01-131	1	18	7	0.013	0.95-0.9	He	1	1	16	1	2.373		0.25-0.1	?
E4H01-273	1	19	6	0.013	0.95-0.9	He	1	1	17	0				Ho
E4H03-253	1	20	5	0.12	0.75-0.5	He	1	0	10	7		0.235	0.75-0.5	Ho
E4H05-091	1	18	6	0.056	0.9-0.75	He	1	0	9	8		0	> 0.995	Ho
E4H05-142	1	18	6	0.056	0.9-0.75	He	1	1	17	0				Ho
E4H05-167	0	0	25			Ho	0	1	13	4		3.765	0.1-0.05	He
E4H05-481	0	0	25			Ho	0	1	6	11		0.941	0.5-0.25	He
E4H09-071	0	14	11	3.85	0.05-0.025	?	0	1	8	9		0	> 0.995	He
E4H09-095	1	25	0			Ho	1	0	17	0				Ho
E4H12-079	0	0	25			Ho	0	1	5	12		2.118	0.25-0.1	He
E4H13-369	0	0	25			Ho	0	1	6	11		0.941	0.5-0.25	He
E4H14-158	1	16	9	1.08	0.5-0.25	He	1	0	12	5		2.118	0.25-0.1	Ho
E4H14-350	1	25	0			Ho	1	0	17	0				Ho
E4H15-124	1	17	8	0.333	0.75-0.5	He	1	1	14	3	0.176		0.75-0.5	He
E4H15-313	1	20	4	0.52	0.9-0.75	He	1	1	16	1	2.373		0.25-0.1	?
E4H15-550	1	4	20	38.92	< 0.005	?	1	1	11	6	0.49		0.5-0.25	He
E4H16-137	1	25	0			Ho	1	0	17	0				Ho

To confirm the heritance of epialleles, band E1H5-486, which was present in TN02 but absent in MD24, was isolated from agarose gel, cloned and sequenced. The regions corresponding to E*coR *I and H*ap *II/M*sp *I sites were sequenced in MD24, TN02, and there were no sequence difference found between them. Bisulphite conversion the genomic DNAs from the parents and the 17 individuals in MT2402 F1pop was conducted. A pair of bisulphite conversion specific primer (Additional file 1) was used to amplify the E1H5-486 region. PCR amplified bands were cloned into pGEM-Teasy vector and twelve randomly chosen colonies containing insert were sequenced around E1H5-486 region at the restriction site of H*ap *II/M*sp *I. For a sample homozygote for the locus, all sequences from 12 colonies would be the same. For a heterozygote sample for the locus, two types of sequence were obtained with each represent one allele. It was found that MD24 was homozygote in the locus with the inner cytosine methylated while TN02 was heterozygote with one allele not methylated. For the progenies, their sequences matched exactly with E1H5-486 band in each plant: absence of the band when the locus is homozygote with inner cytosines methylated and presence of band when the locus is heterozygote with one allele not methylated (Table 6 and Additional file 5).

**Table 6:** Bisulphite sequencing of genomic region of E1H5-486 (ATCCGGTA)

Sample	E1H5-486	Bis-sequencing (allele 1//allele 2)
TN02	1	ATTTGGTA//ATTCGGTA
MD24	0	ATTCGGTA//ATTCGGTA

MT2402-01	0	ATTCGGTA//ATTCGGTA
MT2402-02	1	ATTTGGTA//ATTCGGTA
MT2402-03	0	ATTCGGTA//ATTCGGTA
MT2402-04	0	ATTCGGTA//ATTCGGTA
MT2402-05	1	ATTTGGTA//ATTCGGTA
MT2402-06	0	ATTCGGTA//ATTCGGTA
MT2402-07	1	ATTTGGTA//ATTCGGTA
MT2402-08	1	ATTTGGTA//ATTCGGTA
MT2402-09	1	ATTTGGTA//ATTCGGTA
MT2402-10	0	ATTCGGTA//ATTCGGTA
MT2402-11	1	ATTTGGTA//ATTCGGTA
MT2402-12	1	ATTTGGTA//ATTCGGTA
MT2402-13	1	ATTTGGTA//ATTCGGTA
MT2402-14	0	ATTCGGTA//ATTCGGTA
MT2402-15	1	ATTTGGTA//ATTCGGTA
MT2402-16	0	ATTCGGTA//ATTCGGTA
MT2402-17	0	ATTCGGTA//ATTCGGTA

## Discussion

In this study, jatropha seeds were collected in five countries on three continents. Seeds were brought into Singapore and grown in one single farm under the same agronomic practices in order to reveal difference in heritable agronomic traits. The plants were tracked for many agronomic traits including time to first flowering, accumulated yield in one year and the second year, number of primary branch, number of fruiting branch, diameter of canopy after two years, plant height after two years, dormancy of branch and oil content in seed. These traits are important for agronomy study but seldom studied systematically. Tracking for two years will ensure reliability of data.

With this systematic comparison of populations for two years, agronomic differences that are more genetic dependent and less climate and agronomic practice dependent is confirmed among collections. It should be noted that within population performance is also present, as indicated by big standard deviation (> 15% average) in some traits especially yields. Better field performance of Indonesian population (MD) may suggest its better adaptation to the humid tropical climate in Indonesia and Singapore.

Surprisingly, there was very little genetic diversity among the populations to match with agronomic trait differences we found. AFLP is believed to be a highly polymorphic genotyping technology that can scan the genome for nucleotide sequence difference at and near restriction sites. In our study, almost all the 575 distinct bands (with the exception of only three bands in five individuals) were monomorphic. We repeated the same experiment twice with the same results. To address the concern of insufficient or biased coverage by our fAFLP analysis, we used polymorphic molecular markers reported by others, of multiple technologies including RAPD, DAMD, SSR and SCAR to analyse the same samples. These further studies all confirmed the lack of detectable genetic diversity in the populations studied. There are two possible reasons for this paradox. One is that all the genotyping methods together are not sensitive enough to identify genetic diversity among the collections. Alternatively, epigenetic mechanism that changing phenotype (appearance) or gene expression with no requirement of changing to the underlying DNA sequence is possibly involved.

The lack of genetic diversity prompted us to look into the possible epigenetic diversity. Methylation of DNA is one of the major epigenetic markers which affect gene expression directly or indirectly. DNA methylation sensitive fAFLP (MfAFLP) is a modified version of AFLP [13] using a pair of methylation insensitive/sensitive isoschizomer restriction enzymes to locate methylated AFLP markers. In normal AFLP analysis, a methylation insensitive enzyme M*se *I is used (cutting at 5'-TTAA-3') to digest genomic DNA together with a six nucleotide cutter like E*co*R I before PCR amplification, variation in banding pattern reflects only the sequence difference. In this study, a pair of isoschizomers, H*pa *II and M*sp *I, which recognize the same tetranucleotide 5'-CCGG-3' but with different sensitivity to outer and inner cytosine methylation were used in place of M*se *I. The same genomic DNA sample is digested in two reactions, one with E*co*R I/M*sp *I (EM) and the other with E*co*R I/H*pa *II (EH). Differences in EM and EH amplified bands identify cytosine methylation. CpG methylation (inner cytosine methylation) was found to be the most frequent followed by double cytosine methylation. For these two methylations, there were highly significant differences (p < 0.01) among different collections. Compared to the very low percentage of polymorphic band (0.52%) revealed by fAFLP, epigenetic polymorphic bands ranged from 22.42% to 27.58% in the populations. The AMOVA showed significant diversity both among populations and within populations. Similarly, there are numerous cytosine methylation polymorphisms between rice cultivars [15] and cotton collections[16], and the number of methylation differences is not correlated with their genetic distance. This was also found to be true in *A. thaliana *accessions [17]. Since methylation has been implicated in gene regulation and various development processes in plants [10], epigenetic diversity we found in jatropha may play a role in determining jatropha agronomic traits and its development.

The genetic improvement of plants requires individuals that differ in heritable traits. DNA methylation can generate novel and heritable phenotypic variation by influencing gene expression [10]. Stable epigenetic events might be important for plant breeding. In this study, jatropha plants collected from various parts of the world exhibited different morphology features when they were planted side by side, notably in their seed yields, oil contents, branching pattern, flowering time, as well as plant height, canopy and dormancy of the branches. The different morphology features have been observed to have stable inheritance in the next generation (data not shown). Further research on inheritance of epigenetic markers based on the MfAFLP method in an intra-species hybrid population (MT2402F1pop) and a selfed population (MD24F1Scpop) revealed that most polymorphic epigenetic markers were heritable and conformed to Mendelian segregation pattern. In the near future, we will focus on the development of epigenetic markers which are linked to important traits and use these markers to perform marker-assisted breeding.

In tropical and subtropical countries, jatropha has high potential as a biofuel crop. Among the oil-bearing tree species, jatropha is desired due to its multiple positive attributes including drought hardiness, rapid growth, easy propagation, high oil content, small gestation period, wide adaptation [18] as well as high quality biodiesel that its oil can be converted to. It is, however, not domesticated, as indicated by the highly variable and unpredictable field performance. There is an immediate need to breed elite varieties for plantation. It is widely believed that the key to success of any breeding program is the availability of collections with desired traits and maximum diversity among collections. Phenotypic variation and seed biochemical composition have been widely reported. Genetic diversity, on the other hand, has been found to be lower than previously thought. Here we report the moderate level of epigenetic diversity and heritability of epigenetic markers, which can also be used to evaluate diversity among jatropha collections.

## Conclusions

In summary, we systematically compared collections from five countries on three continents. Seed derived plants were planted in the same farm under the same agronomic practices. Multiple agronomic traits were monitored over a period of two years. Our result confirmed the differences in various agronomic traits among collections as well as within collections. These differences should be determined genetically because of their growth in the same climate under the same practices. Paradoxically, there is lack of genetic diversity to match with agronomic differences. We found moderate level of epigenetic diversity, variation in CCGG methylation pattern and proved that many epigenetic markers are heritable and most epialleles follow Mendelian segregation pattern. All these point to the possible involvement of epigenetics in jatropha development, which needs to be further substantiated.

## Methods

### Plant material

Seeds of jatropha collected from five countries/regions including Yunnan province, China (CN); Java, Indonesia (MD); Tamil Nadu, India (TN); Suriname, South America (SU) and Tanzania, Africa (AF) were germinated and transplanted into pits of 1 m × 1 m × 1 m filled with top soil with space of 2 m × 2 m for each tree on the farm of Temasek Life sciences Laboratory (TLL), Singapore. The field management, such as fertilization, pesticides spraying and weeds controlling, followed the normal practices. Only one or two times of irrigation was conducted in the dry season. One year later from the date of sowing, the plants were pruned at the height of 70 cm from ground. A set of 162 randomly selected plants of jatropha from 5 populations were used for genetic and epigenetic diversity analysis. Ten plants randomly pickup from each population were labeled and monitored over a period of two years from March 2007 to February 2010 for various agronomic traits. One intraspecies hybrid population (MT2402F1pop) and one selfed population from the parent MD24 (MD24Scpop) of jatropha were used to detect the inheritance of epigenetic bands.

### DNA extraction

Total genomic DNA was extracted from young leaves of each plant using the DNeasy plant DNA extraction kit (QIAGen, Singapore) following the manufacture's instruction. DNA concentrations were determined using the ND-1000 spectrophotometer (Nanodrop Technologies, Rockland, Delaware, USA) and the quality was checked by electrophoresis on 0.8% agarose gel.

### fAFLP, MfAFLP, SSR and RAPD analysis

fAFLP analysis was conducted as previously reported [19]. Selective amplification was carried out with 23 different combinations of E*coR *I and M*se *I selective primers, with each gave about 25 distinct bands. DNA methylation-sensitive fAFLP (MfAFLP) is a modified version of fAFLP using a pair of methylation-sensitive restriction enzymes, M*sp *I and H*pa *II. They are a pair of isoschizomers, which recognize the same tetranucleotide 5'-CCGG-3' but have different sensitivity to CpG methylation[14]. M*sp *I and H*pa *II were used to replace the enzyme M*se *I in fAFLP. The structure of M*sp *I-H*pa *II adapter and primers were ordered from Oligo (Singapore) according to Xu (2000) [20]. 14 primer pairs from 54 primer pairs with the most number of amplified bands were used to carry out selective amplification. 0.6 uL PCR products of the amplification were analyzed with ABI 3730 ×l DNA analyzer and data processed by GeneMapper 3.7 (Applied Biosystems, USA).

SSR, RAPD and DAMD analysis followed the reports from Basha et al (2007) [5], Shirish et al (2008) [11] and Sudheer P. D.V.N. et al (2009)[12].

### Data collection and statistical analysis

An AFLP Excel Macro [21] was used to convert allele size data from GeneMapper 3.7 (Applied Biosystems, USA) into binary form, to indicate the presence (1) or absence (0) of alleles. To elucidate the epigenetic diversity within and among populations, we scored the MfAFLP bands by the criterion described in Li et al (2008) [22] with several modifications. Bands presented in both lanes of E*coR *I/M*sp *I (EM) and E*coR *I/H*pa *II (EH) were given a binary genotype of EM/EH (1/1); those bands presented in either M*sp *I or H*pa *II were given a binary genotype of EM/EH (1/0) or EM/EH (0/1); bands absent from both M*sp *I and H*pa *II were given a binary genotype of EM/EH (0/0). GenAlEx 6 [23] was employed to compute allele frequency in populations, genetic heterozygosity within populations and, the pairwise Nei's genetic distance between populations. It was also used to conduct Analysis of Molecular Variance (AMOVA) and Principle Coordinates Analysis (PCoA). The ANOVA analysis for the variation of CCGG methylation among 5 populations was conducted with XLStat (version 7.5.2, Addinsoft, USA), which was also used for multiple group Tukey (HSD) analysis. The same software was also used for ANOVA analysis and Tukey (HSD) analysis of all agronomic traits.

### Sodium bisulphite sequencing

Genomic DNAs from the parent plants and 17 hybrid F1 plants were subjected to sodium bisulphite treatment using EpiTect^® ^Bisulphite kits (QIAGEN, Singapore) following the procedures recommended by the manufacture. We used the Methyl Primer Express^® ^Software v1.0 (Applied Biosystems, USA) for the design of bisulphite primers. AmpliTaq Gold™ DNA Polymerase (Applied Biosystems, USA), which is a highly pure and modified form of Taq DNA polymerase, was used to increase PCR product yield and reduce non-specific amplification. To each PCR tube we added 9.0 μl sterile distilled water, 2.0 μl of GeneAmp 10× PCR Buffer II (500 mM potassium chloride and 100 mM Tris-HCl), 2.0 μl of 25 mM MgCl_2 _solution, 2.0 μl of 2 mM dNTPs solution, 1.0 μl of 10 mM bisulphite converted DNA, 2.0 μl of 10 mM forward BSP (Bisulphite Sequencing Primer) solution and 2.0 μl of 10 mM reverse BSP solution, and 0.2 μl of AmpliTaq Gold™ DNA Polymerase. The PCR steps and cycles were as follows: 95°C for 10 min, 6 cycles of 94°C for 45 sec, 56°C for 2 min, 72°C for 2 min, 31cycles of 94°C for 30 sec, 56°C for 1.5 min, 72°C for 1 min. 0.8 μl of shrimp alkaline phosphatase (SAP, New England BioLabs, USA) and DNA exonuclease (New England BioLabs, USA) were then added to 5 μl of the PCR product. The SAP and PCR product mixture is then put onto the thermal cycler at 37°C for 30 minutes, which is the optimum temperature for activity of SAP. SAP will remove the phosphate groups from the 5' end of the DNA strands. This will prevent back to back ligation of amplified PCR product. The mixtures are then subject to a temperature of 85°C for 15 minutes to denature and inactivate the SAP. PCR amplified bands were cloned into pGEM-T Easy vector and twelve randomly chosen colonies had their inserts amplified with SP6/T7 primers before sequence analysis around E1H5-486 locus at the restriction site of H*ap *II/M*sp *I. For direct DNA sequencing, we utilized a dye-labelled dideoxynucleotide chain termination method, using the Applied Biosystems BigDye^® ^Terminator v3.1 Cycle Sequencing Kits (Applied Biosystems, USA). In each PCR reaction tube, we added 4.0 μl Terminator Ready Reaction Mix, 5.5 μl sterile distilled water, 0.5 μl PCR product, and 0.16 μl of 10 mM SP6 primer for each sequencing reaction.

## Authors' contributions

CXY participated in the design of the study, carried out the field trail, collected data, conducted experiments and drafted the manuscript. SLZ helped the field trail and collected data. XKL conducted fAFLP and bisulphite genomic sequencing experiments. HTNB conducted chemical analysis. YH designed and coordinated the study, participated in data analysis and edited the manuscript. All authors read and approved the manuscript.

## Supplementary Material

Additional file 1 **Details of primers for the study**. Primer ID and sequences Click here for file

Additional file 2 **fAFLP analysis of Jatropha collections**. Restriction enzyme and primer combination E3B Click here for file

Additional file 3 **MfAFLP analysis of Jatropha collections**. Restriction enzyme and primer combination E1H5 Click here for file

Additional file 4 **MfAFLP analysis of Jatropha collections**. Restriction enzyme and primer combination E1M5 Click here for file

Additional file 5 **Bisulphite sequencing result**. Sequences of E1H5-486 locus (bracketed) in parents and progenies of MT2402 F1pop Click here for file

## References

[B1] Fairless D Biofuel: the little shrub that could--maybe Nature 2007 449 7163 652 655 10.1038/449652a 17968401

[B2] Heller J Physic nut. Jatropha curcas L Promoting the conservation and use of underutilized and neglected crops 1 1996

[B3] Dehgan Ba GLW Morphology and infrageneric relationships of the genus Jatropha (Euphorbiaceae) 1979 74 University of California Publications in Botany

[B4] Kaushik N Krishan K Sushil K Nutan K Roy S Genetic variability and divergence studies in seed traits and oil content of Jatropha (Jatropha curcas L.) accessions Biomass and Bioenergy 2007 31 7 497 502 10.1016/j.biombioe.2007.01.021

[B5] Basha SD Sujatha M Inter and intra-population variability of Jatropha curcas L. characterized by RAPD and ISSR markers and development of population-specific SCAR markers Euphytica 2007 156 3 375 386 10.1007/s10681-007-9387-5

[B6] Basha SD George F Makkar HPS Becker K Sujatha M A comparative study of biochemical traits and molecular markers for assessment of genetic relationships between Jatrpoa curcas L. germplasm from different countries Plant Science 2009 176 6 812 823 10.1016/j.plantsci.2009.03.008

[B7] Popluechai S Breviario D Mulpuri S Makkar HPS Raorane M Reddy AR Palchetti E Gatehouse AMR Syers JK O'Donnell AG Narrow genetic and apparent phenetic diversity in Jatropha curcas: initial success with generating low phorbol ester interspecific hybrids Nature Precedings 2009 (10101/npre.2009.2782.1)

[B8] Henderson IR Jacobsen SE Epigenetic inheritance in plants Nature 2007 447 7143 418 424 10.1038/nature05917 17522675

[B9] Doerfler W DNA methylation and gene activity Annu Rev Biochem 1983 52 93 124 10.1146/annurev.bi.52.070183.000521 6311083

[B10] Lukens LN Zhan SH The plant genome's methylation status and response to stress: implications for plant improvement Current Opinion in Plant Biology 2007 10 3 317 322 10.1016/j.pbi.2007.04.012 17468039

[B11] Shirish AR Srivastava AP Rana TS Srivastava J Tuli R Easy assessment of diversity in Jatropha curcas L. plants using two single-primer amplification reaction (SPAR) methods Biomass and Bioenergy 2008 32 6 533 540 10.1016/j.biombioe.2007.11.006

[B12] Sudheer Pamidiamarri DVN Sinha R Kothari P Reddy MP Isolation of novel microsatellites from Jatropha curcas L. and their cross-species amplification Molecular Ecology Research 2009 9 1 431 433 10.1111/j.1755-0998.2008.02471.x 21564671

[B13] Vos P Hogers R Bleeker M Reijans M van de Lee T Hornes M Frijters A Pot J Peleman J Kuiper M AFLP: a new technique for DNA fingerprinting Nucleic Acids Res 1995 23 21 4407 4414 10.1093/nar/23.21.4407 7501463 PMC307397

[B14] Reyna-López GE Simpson J Ruiz-Herrera J Differences in DNA methylation patterns are detectable during the dimorphic transition of fungi by amplification of restriction polymorphisms Molecular and General Genetics 1997 253 6 703 710 10.1007/s004380050374 9079881

[B15] Ashikawa I Surveying CpG methylation at 5'-CCGG in the genomes of rice cultivars Plant Molecular Biology 2001 45 1 31 39 10.1023/A:1006457321781 11247604

[B16] Keyte AL Percifield R Liu B Wendel JF Infraspecific DNA methylation polymorphism in cotton (Gossypium hirsutum L.) J Hered 2006 97 5 444 450 10.1093/jhered/esl023 16987937

[B17] Cervera MT Ruiz-Garcia L Martinez-Zapater JM Analysis of DNA methylation in Arabidopsis thaliana based on methylation-sensitive AFLP maekers Mol Genet Genomics 2002 268 2 543 552 10.1007/s00438-002-0772-4 12471452

[B18] Sujatha M Reddy TP Mahasi MJ Role of biotechnological interventions in the improvement of castor (Ricinus communis L.) and Jatropha curcas L Biotechnol Adv 2008 26 5 424 435 10.1016/j.biotechadv.2008.05.004 18579331

[B19] Hong DY Lau AJ Yeo CL Liu XK Yang CR Koh HL Hong Y Genetic diversity and variation of saponin contents in Panax notoginseng roots from a single farm J Agric Food Chem 2005 53 22 8460 8467 10.1021/jf051248g 16248538

[B20] Xu ML Li XQ Korban KS AFLP-based detection of DNA methylation Plant Molecular Biology Reporter 2000 18 4 361 368 10.1007/BF02825064

[B21] Rinehart TA AFLP analysis using Genemapper software and an Excel Macro that aligns and converts output to binary Biotechniques 2004 37 2 186 188 15335205 10.2144/04372BM01

[B22] Li YD Shan XH Liu XM Hu LJ Guo WL Liu B Utility of the methylation-sensitive amplified polymorphism (MSAP) marker for detection of DNA methylation polymorphism and epigenetic population structure in a wild barley species (hordeum brevisubulatum) Ecological Research 2008 23 5 927 930 10.1007/s11284-007-0459-8

[B23] Peakall R Smouse PE Genalex 6: genetic analysis in Excel. Population genetic software for teaching and research Molecular Ecology Notes 2006 6 1 288 295 10.1111/j.1471-8286.2005.01155.x PMC3463245 22820204

