# Supplementary material for: Does epigenetic polymorphism contribute to phenotypic variances in Jatropha curcas L.?
Source: BMC Plant Biol. 2010 Nov 23;10:259. doi: 10.1186/1471-2229-10-259 (PMC3017842; doi:10.1186/1471-2229-10-259)

Additional file 2: fAFLP analysis of Jatropha collections with restriction enzyme and primer combination E3B

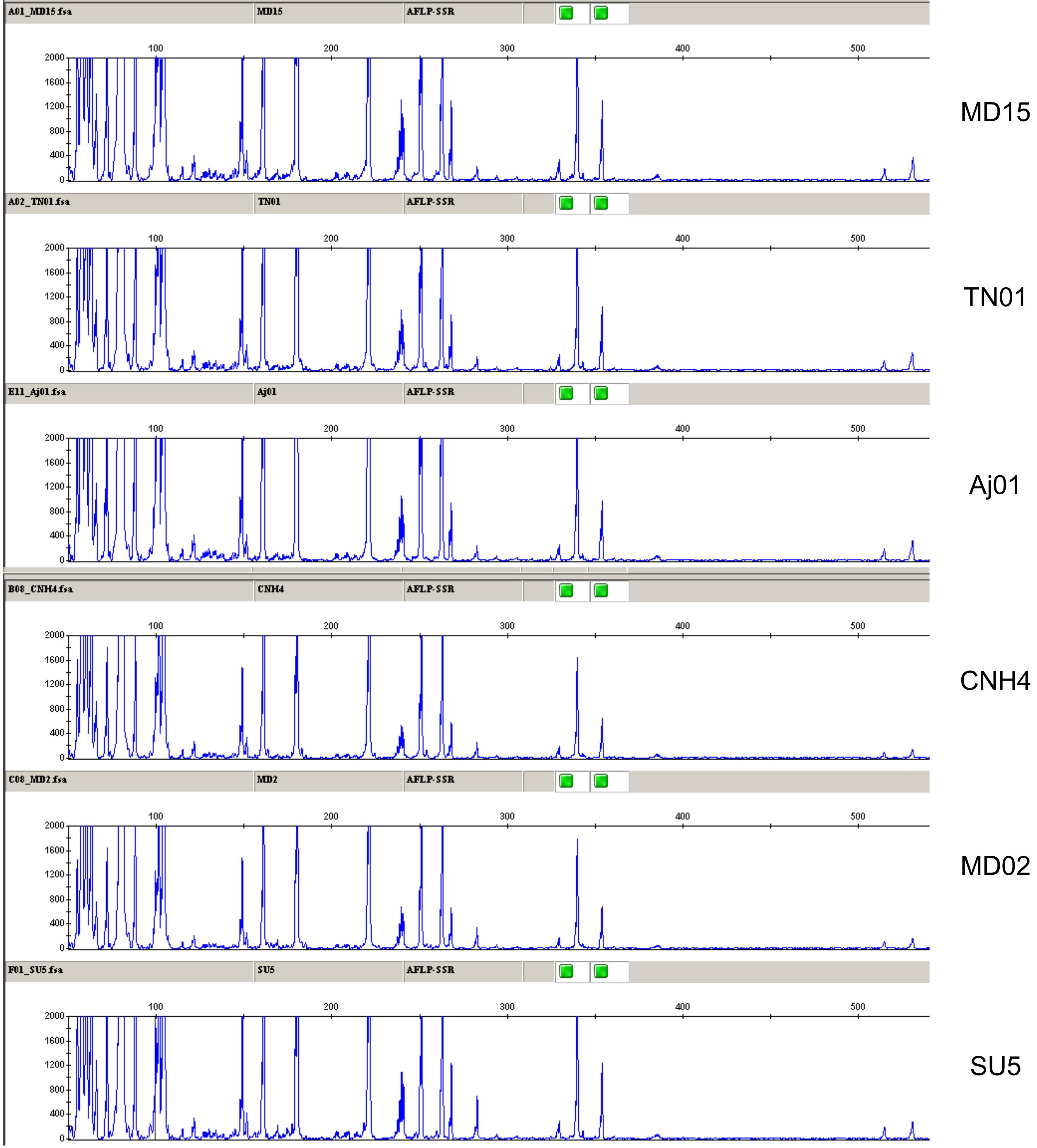

Supplement: Additional file 2 — fAFLP analysis of Jatropha collections. Restriction enzyme and primer combination E3B [file 1471-2229-10-259-S2.PDF]
