# Supplementary material for: Does epigenetic polymorphism contribute to phenotypic variances in Jatropha curcas L.?
Source: BMC Plant Biol. 2010 Nov 23;10:259. doi: 10.1186/1471-2229-10-259 (PMC3017842; doi:10.1186/1471-2229-10-259)

Additional file 5: Bisulphite sequencing of E1H5-486 locus (bracketed) in parents and progenies of MT2402 F1pop

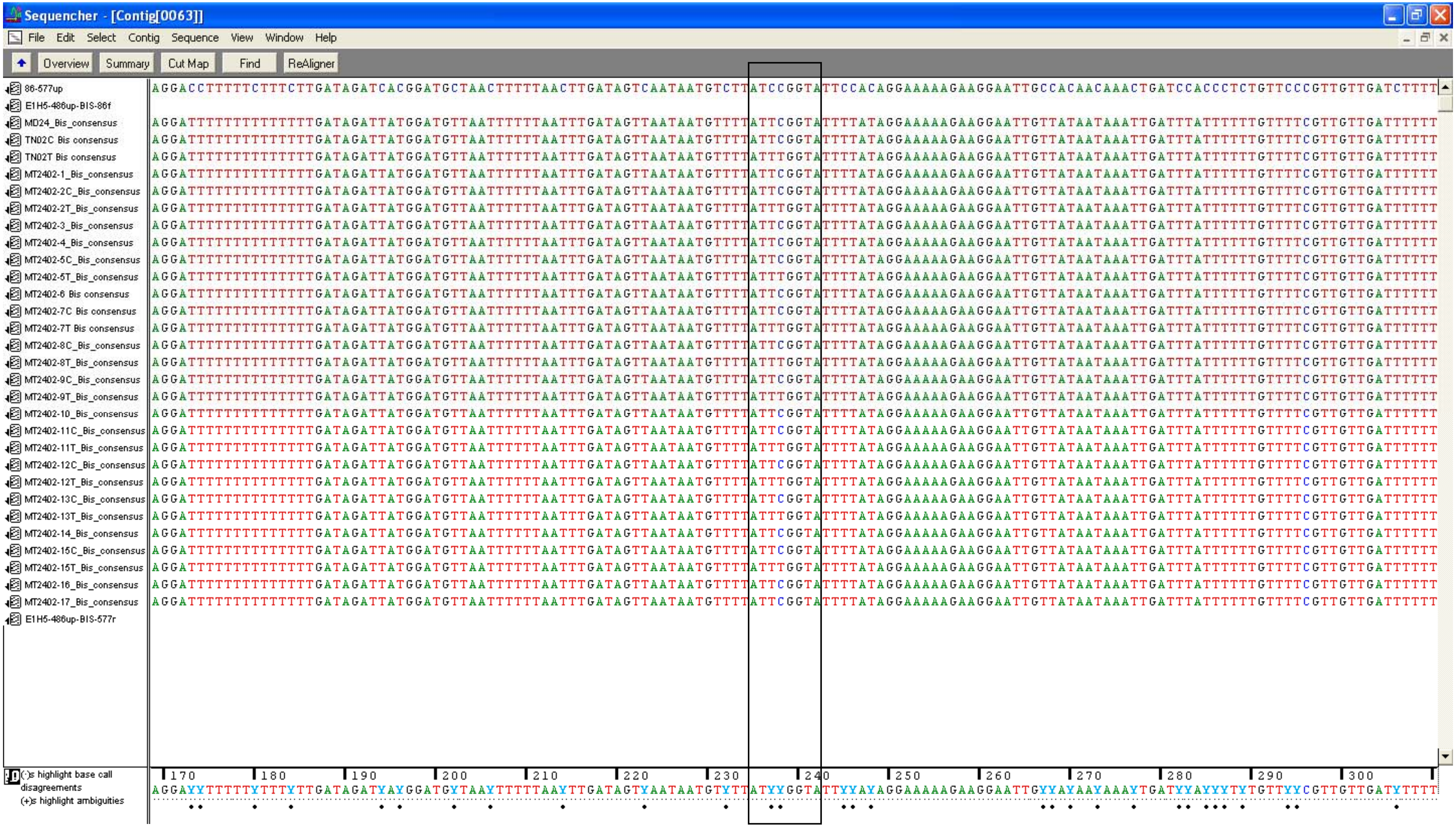

Supplement: Additional file 5 — Bisulphite sequencing result. Sequences of E1H5-486 locus (bracketed) in parents and progenies of MT2402 F1pop [file 1471-2229-10-259-S5.PDF]
